# Domestic Dog Scent Marks Trigger a Behavioural Response in Wild Wolves

**DOI:** 10.1002/ece3.71364

**Published:** 2025-07-09

**Authors:** Kinga M. Stępniak, Tom A. Diserens, Maciej Szewczyk, Robert W. Mysłajek, Dries P. J. Kuijper

**Affiliations:** ^1^ Department of Animal Ecology and Evolution, Institute of Ecology Faculty of Biology University of Warsaw Warsaw Poland; ^2^ Mammal Research Institute Polish Academy of Sciences Białowieża Poland; ^3^ Faculty of Biology University of Warsaw Warsaw Poland; ^4^ Department of Vertebrate Ecology and Zoology, Faculty of Biology University of Gdańsk Gdańsk Poland

**Keywords:** chemical communication, conservation, intraspecific interactions, territory defence

## Abstract

The European grey wolf ( *Canis lupus* ) and domestic dog ( *Canis lupus familiaris* ) share not only a common origin but also many similarities in behaviour. Due to the introduction of legal protection, wolves have recolonised large parts of Europe. They are increasingly inhabiting human‐dominated landscapes, leading to a growing potential for interactions between wolves and domestic dogs. However, these interactions between wolves and dogs are still poorly understood. Scent marking is one of the main forms of communication in canids and is crucial for marking territories, synchronising reproduction, establishing hierarchies within groups, and forming new breeding pairs. We hypothesised that the presence of domestic dog scent markings in wolf territories may induce a behavioural response and therefore interfere with wolf behaviour. To test this, we experimentally scent‐marked objects within known wolf home ranges in Kampinos National Park, Poland, to simulate the presence of “unknown dogs” (dog urine from outside the area) and water as a control. To test whether and how wolves' behavioural response to the scents of domestic dogs and wolves differs, we additionally left scent marks of “unknown wolves” (wolf urine from outside the area). Using camera traps, we studied the behavioural responses of local wolf families exposed simultaneously to all three scent stimuli. Juveniles spent significantly more time sniffing wolf scent (37.1 ± 8.9 s) than dog scent (7.1 ± 3.4 s), while breeding pairs displayed more diverse marking behaviours, including overmarking and ground scratching, particularly in response to wolf scent. Wolves spent a longer time responding to wolf scent marks than to dog scent marks, indicating they may distinguish between them, but inexperienced juveniles spent much more time exploring dog scent marks than adults. Our results indicate that domestic dog scent marks trigger a behavioural response in wild wolves. This suggests that the increasing occurrence of dogs inside wolf territories could affect and potentially disturb the scent‐marking behaviour of wolves.

## Graphical abstract

Wolves and Dogs Coexist in Human‐Dominated Landscapes: As wolves recolonise parts of Europe, they increasingly encounter domestic dogs, leading to interactions that are poorly understood, especially in terms of scent marking and communication. Scent Marking Experiment: In an experiment in Kampinos National Park, Poland, wolf territories were scent‐marked with urine from “unknown dogs” and “unknown wolves” to observe behavioural responses via camera traps. Water was used as a control. Wolves Respond More to Wolf Scent Marks: Wolves spent significantly more time sniffing scent marks from “unknown wolves” compared to “unknown dogs”, indicating that wolves may differentiate between these scents and prioritise responses to conspecifics. Age‐Related Differences in Behaviour: Juvenile wolves spent more time exploring dog scent marks than adult wolves, suggesting inexperience or greater curiosity. Adults primarily focused on wolf scent marks, displaying behaviours like overmarking and ground scratching. Implications for Wolf Behaviour: The presence of domestic dogs in wolf territories could interfere with wolves' natural scent‐marking behaviours, especially for juveniles. These findings highlight the need for further research on how dog presence might disrupt wolf territoriality and communication.

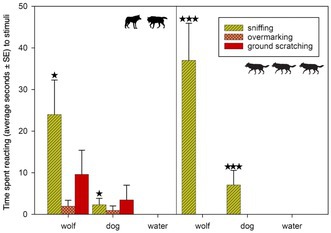

## 1 Introduction

Strict legal protection has led to an increase in the ranges and numbers of wolf (*Canis lupus*) populations across Europe (Chapron et al. 2014; Nowak and Mysłajek 2016). The return of wolves to human‐dominated landscapes often puts them into contact with “humans' best friend”, domestic dogs (*C. l. familiaris*). In many places around the world, wolves and dogs live close to each other or even share the same landscape (Wierzbowska et al. 2016; Mysłajek et al. 2018, 2022; Salvatori et al. 2020) in which the wild and domesticated forms of the same species have the opportunity to meet and interact. This raises questions about whether dogs and wolves interact and what the consequences of these interactions are for wild wolf behaviour.

Wolves and dogs can interact in several ways. They are able to interbreed and produce fertile offspring (Salvatori et al. 2020). Dogs can also compete with wolves for food resources (Hughes and Macdonald 2013). Moreover, dogs and wolves share many parasites and diseases (Lescureux and Linnell 2014). Among the many pathogens affecting dogs, only a few are believed to be of concern for wolf conservation, but the presence of these in the wild can have severe consequences (Knobel et al. 2014). As a consequence of these impacts, there is a growing concern among conservationists about the negative impacts of dogs on the behaviour and ecology of the wolf (Lescureux and Linnell 2014).

While competitive interactions and parasites have been widely studied, the behavioural response of wolves to the presence of dogs within their territories has generally been overlooked. Dogs and wolves display similar behaviour, even though in some dog breeds it has been distorted by selective breeding during domestication. Scent marking is a primary form of communication in canids, including wolves (Peters and Mech 1975; Dunbar 1977; Allen et al. 1999; Pal 2003; Zub et al. 2003; Stępniak et al. 2020). Canids scent mark using faeces, urine, and interdigital gland secretions left during scratching the ground (Harrington and Asa 2003). Similarly to wolves, dogs also use scent markings to communicate with conspecifics (Scott and Fuller 1965; Pal 2003). Scent marks contain a rich source of information that can greatly affect the behaviour of both dogs and wolves. In wolves, they convey information essential for demarcating territories, reproduction, establishing hierarchies in groups, and the formation of new breeding pairs (Asa et al. 1985; Paquet and Fuller 1990; Paquet 1991; Vila et al. 1994). It also carries a range of information about the individual that left them, e.g., regarding sex, health, or position in the group (Zala et al. 2004; Wyatt 2014).

Scent marking has a much lower cost than attacking or being attacked by an intruder, making it a cost‐effective strategy for protecting a wolf's territory and its resources from potential intruders (Gosling 1982, 1986; Gosling and Roberts 2001). The intensity of an individual scent mark varies seasonally and is influenced by its status (Peters and Mech 1975; Asa et al. 1990). Wolves particularly increase the frequency of their scent marking near the core areas of their territories during pup rearing (Zub et al. 2003; Llaneza et al. 2014). To enhance the chance of detection, wolves often leave their scent marks in conspicuous places like forest road intersections, mostly on conspicuous surfaces and objects (Zub et al. 2003; Stępniak et al. 2020), similarly to free‐living dogs (Cafazzo et al. 2012).

When wolves and dogs occur in the same area, it is likely that they interact via scent marking. However, what information they transfer and how this affects wolf behaviour is as yet unknown. Wolves may perceive the presence of dogs as an indication of risk from humans (Suraci et al. 2019). They may also see dogs as competitors (Hughes and Macdonald 2013). In both cases, wolves should rather avoid dogs or their scent marks (Gosling and Roberts 2001), and they could increase perceived risk (for either humans or dogs). Alternatively, dogs may be seen as potential reproductive mates (Lescureux and Linnell 2014), which could lead to wolves being attracted to dogs or their scent marks. In light of these potential impacts and the increasing presence of wolves in human‐ and dog‐dominated landscapes in Europe, whether and to what extent wolves and dogs communicate with each other via scent marks and how this alters wolf behaviour is therefore an important knowledge gap.

We hypothesise that the presence of domestic dog scent markings in wolf territories may interfere with natural wolf behaviour. To test whether and to what extent wolves react to dog scent marks and how their response differs from reactions to wolf scent marks, we experimentally scent‐marked locations to simulate the presence of “unknown dogs” (dog urine from outside the area) and “unknown wolves” (wolf urine from outside the area), using water as a control. With camera traps, we studied the behavioural response of wolf families living near humans and dogs in an area intensively visited by people (Kampinos National Park, Poland; KNP hereinafter).

## 2 Methods

Our experiment was carried out between 03 February 2020 and 24 March 2020.

### 2.1 Study Area

**FIGURE 1:**
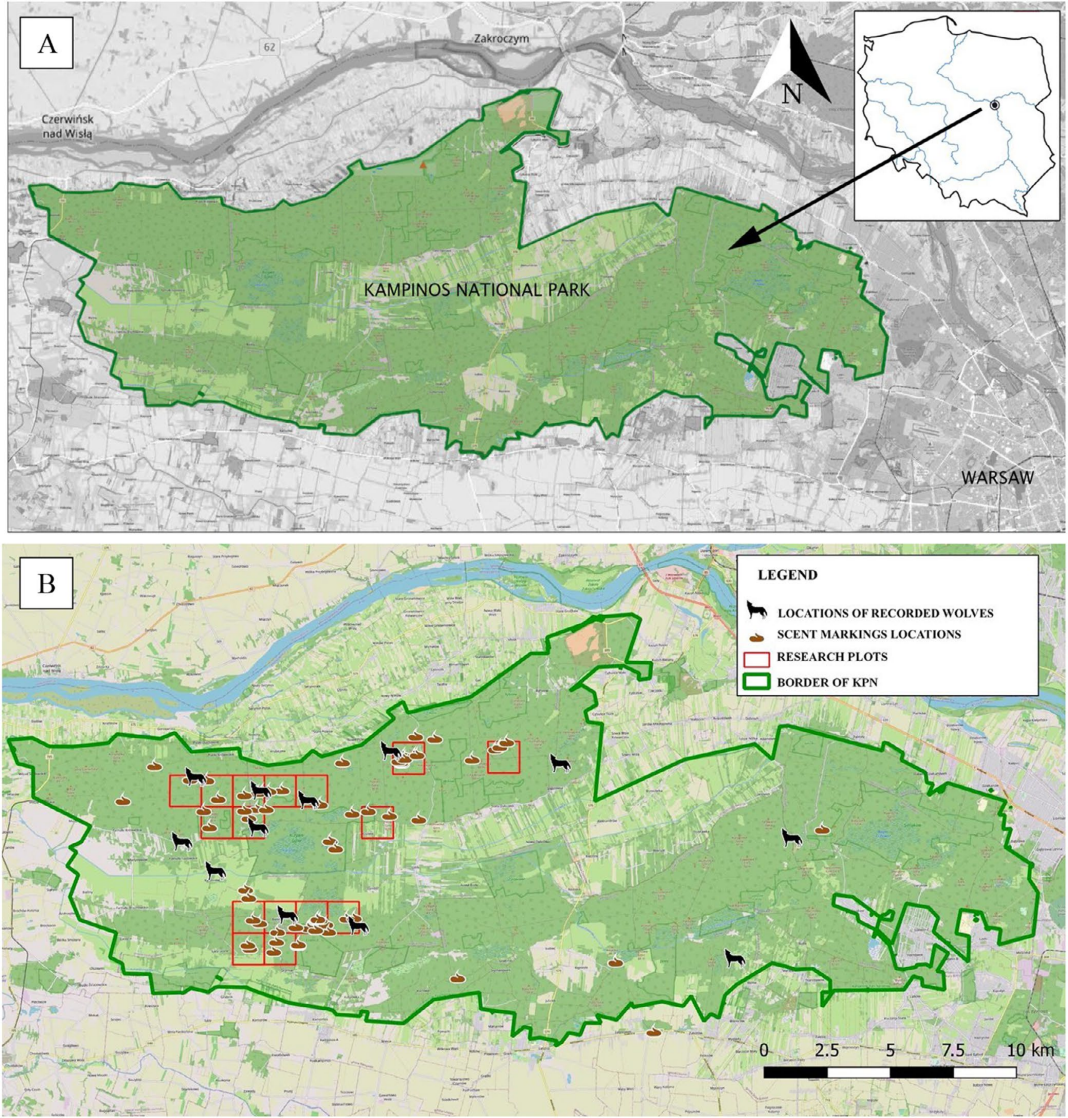
(A) Study area, KNP. (B) Distribution of research areas, scent markings, and locations where wolves were recorded.

KNP was established in 1959. Its total area is 384.7 km^2^, and the buffer zone around the park covers an additional 377.6 km^2^. The area is also protected as the Natura 2000 site “Puszcza Kampinoska” (PLC140001), which covers 376.4 km^2^. In 2000, KNP and its buffer zone were designated as a Biosphere Reserve within the UNESCO‐MaB (Man and Biosphere) programme. The landscape of the park formed in the post‐glacial period and comprises marshes and dunes. KNP encompasses Kampinos Forest in the Vistula proglacial river valley, to the NW part of Warsaw, between the Bzura River and the left bank of the Vistula River (Figure 1A). Forests cover over 73% of the park's area, with Scots pine ( *Pinus sylvestris* ) being the dominant species (70%), followed by black alder ( *Alnus glutinosa* ) (12.5%) and oak (*Quercus* ssp.) (10.3%).

The ungulate community comprises roe deer ( *Capreolus capreolus* ) (78% of total ungulate numbers), red deer ( *Cervus elaphus* ) (9%), moose ( *Alces alces* ) (8%), and wild boar ( *Sus scrofa* ) (5%) (Central Statistical Office 2019). In 1994, the Eurasian lynx ( *Lynx lynx* ) was reintroduced to the park (Boer et al. 2000) and continues to occur there (Mysłajek et al. 2019). The wolf became strictly protected in Poland in 1998 (Nowak et al. 2017), after which the species' range increased and it recolonised KNP. The first confirmed reproduction of wolves in KNP occurred in 2015. Over the study period, two wolf packs lived in KNP, and their total number was estimated at approximately. 12 individuals (K.M. Stępniak, M. Szewczyk, I. Kwiatkowska, R.W. Mysłajek, S. Nowak unpublished data).

KNP, due to its location right next to Warsaw (Poland's capital city), is a very popular recreational destination for residents. Human settlements surround KNP's borders, and some villages lie within the park boundaries. The park, therefore, experiences heavy anthropopressure. Moreover, as the presence of dogs in protected areas is correlated with human presence (Soto and Palomares 2015), these canids are highly abundant in KNP.

Polish law dogs only permit dogs to enter forests on a leash (art. 30, Journal of Laws 2022.0.672 i.j.—Act of 28 September 1991 on forests). The regulations of KNP prohibit dogs from entering, except for assistance dogs (ordinance no. 15/2020 of the Director of KNP dated 31.07.2020 on the improvement of KNP).

### 2.2 Selection of Sample Plots

We located our sample plots in areas with the highest chance of wolf presence as determined from data collected during two previous research projects in KNP:

1. Data from camera traps installed in dense networks at 68 fixed locations in KNP between 03.05.2018 and 28.06.2018 for a total of 2366 camera trap days (unpublished data. results Diserens et al.). Camera traps were deployed along forest roads, on trees at a height of up to 1 m, in places where there was at least 20 m visibility (following Bubnicki et al. 2019),
2. Data from 4 years (2016–2020) of winter wolf tracking in KNP. During each tracking, we collected non‐invasive genetic samples (i.e., scats and urine), noted their location using a hand‐held GPS receiver (Garmin GPSMAP 64 s), and then entered the coordinates into a database (following Szewczyk et al. 2019, Stępniak and Kwiatkowska unpublished data).

Using QGIS (QGIS.org 2021), we plotted a regular grid with 1.25 km^2^ cells on a map of KNP, together with all locations with wolf presence based on these two previous studies. As areas of high wolf activity, we selected cells where wolves were observed on two or more occasions (Figure 1B). Within these cells, we designated plots for the field experiment at intersections (Barja et al. 2004; Stępniak et al. 2020) with scent markings indicating recent wolf presence and sufficient trees for installing camera traps with full visibility over the experimental plots.

All experimental plots were placed in the territory of a single wolf pack in KNP (Figure 1B). During our analysis of the data collected during the experiment, we observed that both wolf packs in the area visited our experimental plots and reacted to the presented stimuli. We could distinguish these two packs based on physical appearance. Because the time of the study was also the period when puppies from the previous litter accompanied their parents, individuals were recognised when the whole pack was recorded, based on differences in the appearance of the reproducing male and female. Due to the nature of the recordings and overlapping appearances, no attempt was made to count individual wolves to avoid overestimation. Differences in wolf behaviour according to family were not included in the statistical analysis.

### 2.3 Study Design

**FIGURE 2:**
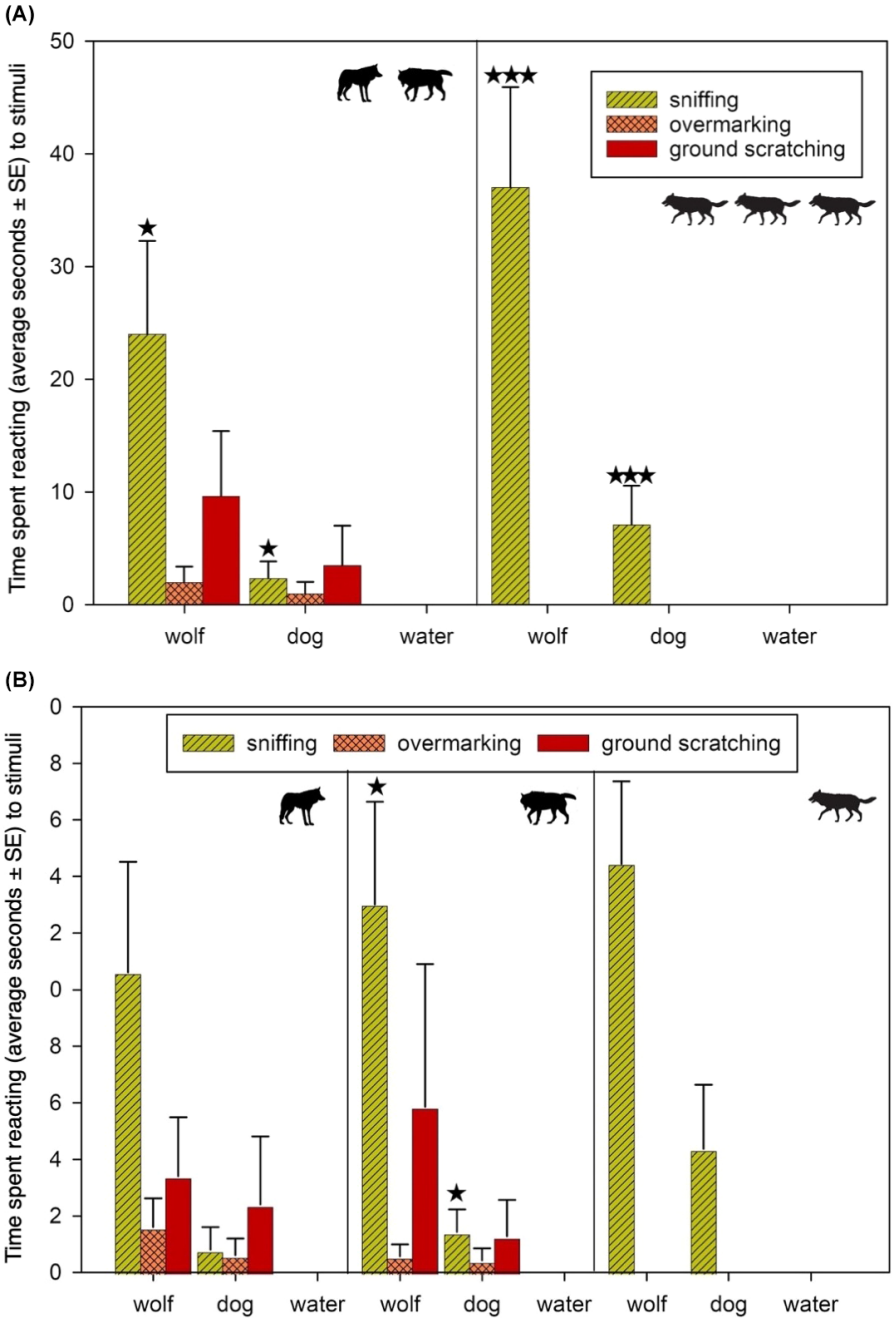
(A) Time spent reacting (average seconds, ± SE) to different stimuli (urine of a unknown wolf, urine of unknown dog, water) by breeding pair (left) and juveniles (right) in KNP. Asterisks indicate levels of statistical significance: *p < 0.05, **p < 0.01, *** *p* < 0.001. “wolf” means unknown wolf, “dog” means unknown dog. (B) Time spent reacting (average seconds, ± SE) to different stimuli (urine of unknown wolf, unknown dog, water) by a breeding female (left), a breeding male (center), and a juvenile (right) in KPN. Asterisks indicate levels of statistical significance: *p < 0.05, **p < 0.01, *** *p* < 0.001. “wolf” means unknown wolf, “dog” means unknown dog.

We applied three treatments at once, at each location at three different plots: (1) dog urine, (2) wolf urine, and (3) water as a control (Figure 2). These treatments were followed for one week. For each following locations, we used urine from a new individual wolf and dog (“unknown wolf” and “unknown dog”, see details under ‘*Wolf and dog urine collection*’) to mimic new individuals appearing in the wolf territory. We replicated this set of three treatments 29 times (hence at 29 different locations across the study area). At each treatment plot, we added 10 mL of urine or water at a height ≤ 0.5 m from the ground. To prevent contamination between individuals' scents, we applied treatments at least 1 m apart, and every time we used a new syringe for each scent stimulus. To reduce contamination with human scent while applying treatments, we wore nitrile gloves. To mimic natural scent marking, all three types of scent stimuli were applied to distinctive elements in the environment (trees, clumps of grass, tree trunks, bushes, etc.). The placement of each scent stimulus at each location (i.e., road, forest crossroads) was fully randomised using a number generator. This ensured that the spatial arrangement of stimuli varied across plots, eliminated systematic positional bias, and prevent wild wolves from always being presented with one of the stimuli first.

To record the wolf behavioural response to scent stimuli, we installed a camera trap (Ltl Acorn 5310 MG or BUSHNELL Trophy cam HD) on trees at a height ≤ 0.5 m from the ground with an unobscured view towards the location of the applied scent, at 8–10 m away. The camera traps were set to record 60 s videos after triggering. The interval between subsequent recordings was set at 1 s. To minimise the effects of ageing of scent stimuli, we set the time of the experiment for 7 days. Once wolves were recorded within this period, we moved to a new location. If no wolves were recorded, we continued at the same location and refreshed scent marks, using the same urine samples from the same individual dog/wolf as in the previous week.

As the response to a new dog scent mark could have been potentially triggered or modified by the nearby presence of a new wolf scent mark, we chose an additional four locations as treatment plots. At these locations, only two treatments were placed: urine from an unknown dog and water. This allowed us to test whether the response towards a new dog is different with and without the presence of a new wolf scent mark. We selected these plots in the same way as those described above.

### 2.4 Wolf and Dog Urine Collection

In the experiment, we used urine from five wolves (two females and three males) and six dogs (three females and three males). Wolf urine was collected from wolves at the Wolf Science Center of the Veterinary University of Vienna. The wolves there are kept under constant veterinary care, vaccinated, and free of internal and external parasites. Urine was collected by wolf trainers directly from the wolves during spontaneous urination into sterile plastic containers and stored frozen. We collected domestic dog urine from six individuals (four females and two males). They were healthy, vaccinated, and dewormed, living in private homes. Urine was collected directly from the dogs during spontaneous urination into sterile plastic containers and stored frozen.

All urine was stored in a −20°C freezer for no more than 18 months. The use of frozen urine is an accepted and commonly used research method (Lisberg and Snowdon 2009). Frozen urine samples were placed in a portable car refrigerator and transported to the field site, where they gradually thawed during transit over approximately 2 h. Once thawed, the urine was not frozen again.

Permission for the study was granted by the director of KNP. In accordance with Polish Law on the Use of Animals in Scientific Experiments, approval from an ethical commission is not required for studies that only indirectly interact with animals.

### 2.5 Video Classification

During the video analysis, we tracked the duration of time that a specific wolf was visible on the footage. Behavioural responses were defined as any clear investigatory behaviour directed towards one of the scent stimuli. We distinguished the following behavioural responses: sniffing, overmarking (with urine), and ground scratching. In contrast, wolves that did not react to the stimulus simply passed through the field of view without stopping, sniffing, or marking—this behaviour was classified as natural behaviour. Such movement could include patrolling, transporting prey or pups, or engaging in other routine activities, especially in the case of the breeding pair. This distinction allowed us to clearly separate deliberate reactions to stimuli from incidental presence on camera. Information about behaviour was noted on an Excel sheet, along with the duration for each type of behaviour.

**FIGURE 3:**
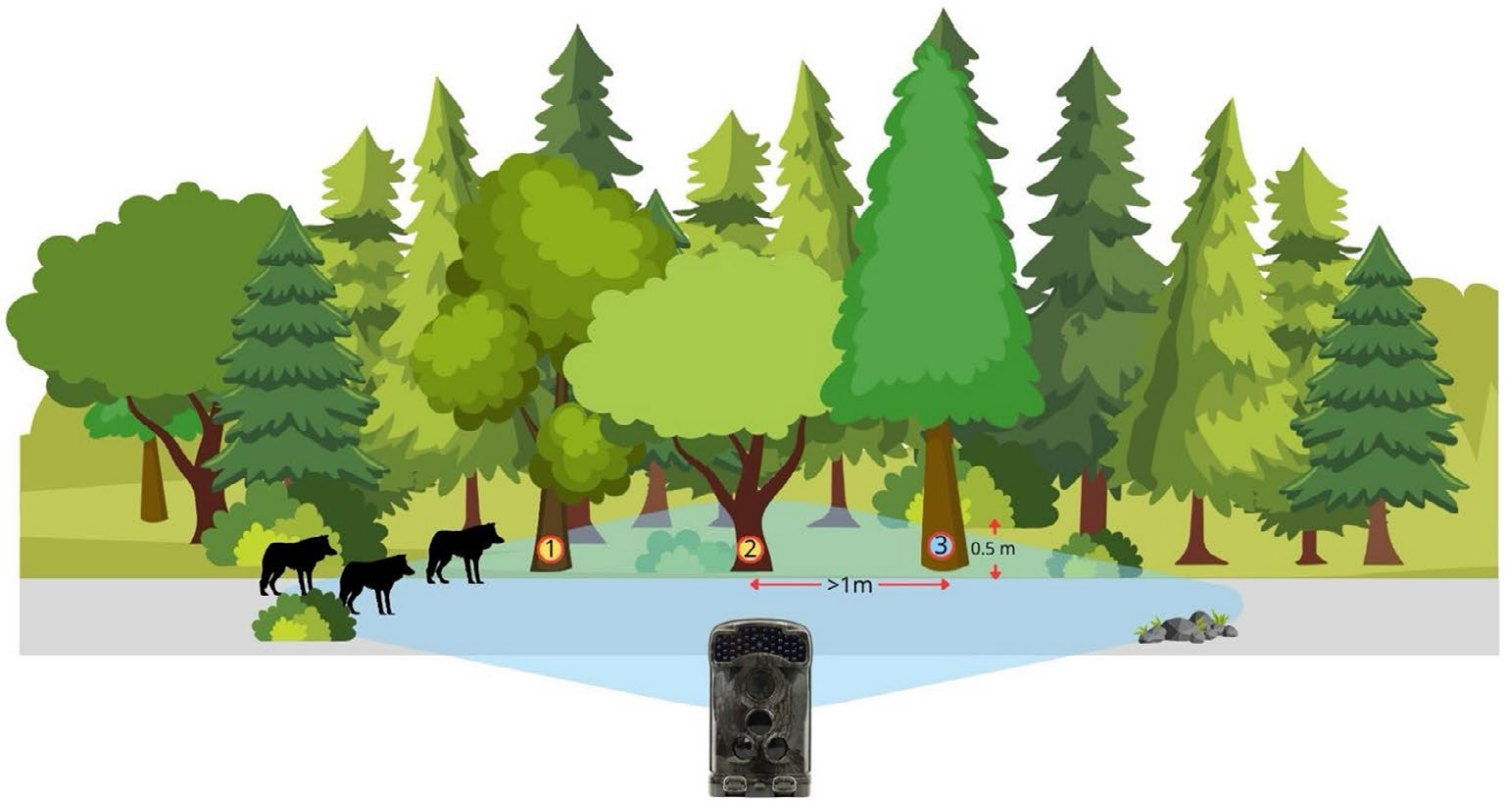
Schematic representation of the spatial layout of the experimental plots, illustrating the placement of scent stimuli (1 = dog urine, 2 = wolf urine, 3 = water) and the positioning of the camera trap. All stimuli were placed at approximately 0.5 m height on natural objects (e.g., trees), with a minimum of 1 m spacing between them to avoid cross‐contamination. The blue polygon indicates the field of view of the camera trap positioned at approximately 8–10 m distance from the stimuli. The placement of each scent stimulus at natural objects was fully randomised using a number generator.

**FIGURE 4:**
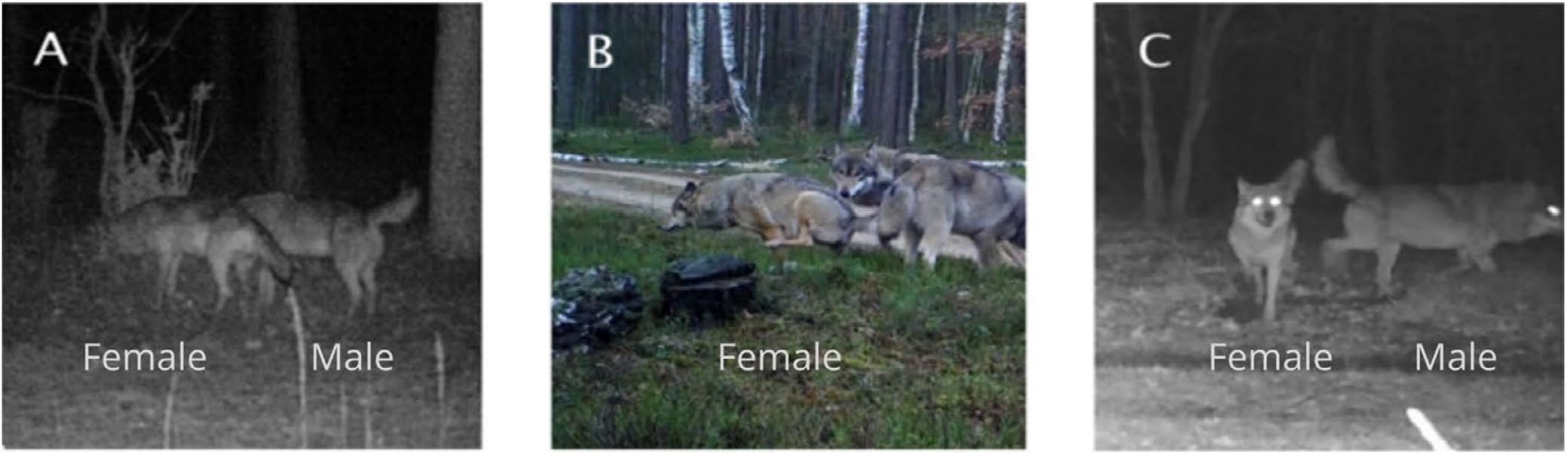
A, B, C Identification of sex in wild wolves based on physical and behavioural characteristics captured by camera traps in Kampinos National Park (A–C).

We classified the videos in the software “Trapper” (Bubnicki et al. 2016). Details of the classification criteria are shown in Table 1. We determined the reproductive status and sex of recorded wolves by assessing them following Goodman et al. 2002. A wolf was placed into a given category (breeding pair or juvenile) based on the following factors: the size of the wolf relative to other pack members, posture, tail position, and behaviour. Members of a breeding pair were usually clearly larger than subadults, carried their tails high as a sign of social dominance, and had a dominant body posture. Secondary sex characteristics and posture during stimulus overmarking were used to discriminate between the sexes in the breeding pair. Males overmark by lifting a raised leg while urinating, whereas females urinate by lifting a bent hind leg and squatting (Peters and Mech 1975). Although posture during urination is generally not a fully reliable sex indicator, in our study, the high‐quality camera trap footage allowed us to clearly observe the distinct urination postures of adult wolves. Therefore, we are confident that the sex determination based on these behavioural criteria was accurate for the individuals analysed (Figure 3 and Figure 4A,B,C).

**TABLE 1:** Shows the classifiers used to analyse camera trap recordings.

Classification category	Classification criteria
The type of recording	Observation, blank, camera trap inspection, recording error
The number of individuals visible on the recording	
Species	Wolf, dog with a human on a leash, dog with a human but not on the leash, dog without a human present
Status (classification only for wolves)	Breeding female, breeding male, juvenile, unspecified
Reaction to the scent stimulus?	Yes, no
Type of stimulus to which the reaction was recorded	Wolf, dog, water
Type of reaction to the scent stimulus	Sniffing, overmarking, ground scratching
Reaction time	Counted in seconds
Time present on the recording	Counted in seconds
Was the stimulus overmarked by another animal?	Yes, no
The species of animal that overmarked the stimulus	Fox, dog, badger, wolf

### 2.6 Statistical Analysis

We performed analysis for different groups: all individually scored wolves combined, all individually scored breeding individuals combined, all individually scored juveniles combined, all individually scored breeding males combined, all individually scored breeding females combined, and single juvenile. The single juvenile was calculated as the average reaction from all the reactions of juveniles and called by name “mean juvenile”.

We analysed wolf behaviour in Microsoft Excel by calculating the percentage of time that an individual wolf spent on behavioural reactions to particular scent stimuli. Then we combined all the results associated with specific groups and calculated the mean value and standard error. By the name “visit duration”, we mean the time when individuals were visible on recordings. The results were visualised in SigmaPlot. We determined the statistical significance of the results in R (R Core Team 2021) using two‐factor ANOVA and Tukey‐HSD tests.

### 2.7 Controls

Throughout the study period, 63 visits by domestic dogs were recorded at the experimental stations. In 31 of these visits, dogs interacted with the scent stimuli, primarily through sniffing. If a dog appeared at any plot and approached the experimental stimuli, the entire site was immediately replaced with a new one to prevent contamination. In cases where dogs overmarked the scent sources and wolves later responded to those locations, such wolf responses were also excluded from the analysis. This ensured that all analysed wolf behaviour reflected reactions to the original, uncontaminated stimuli.

Due to the small number of breeding adults per sex (*n* = 2), comparison between males and females is considered exploratory and should be interpreted with caution.

### 2.8 Ethical Note

Urine samples collected for testing were collected during urination. They were not related to any procedure, so any form of use of animals for specific purposes: in Art. 3, Act of January 15, 2015, on the protection of animals used for scientific purposes or educational purposes, which may cause the animal pain, suffering, distress, or permanent damage to the body. Therefore, no approval from an ethical commission is required. No extra animal discomfort was caused by sample collection for the purpose of this study.

We obtained permission from the Minister of the Environment to disturb vertebrate animals intentionally in the Kampinos National Park (Article 15, section 1, point 3, Act of April 16, 2004, on nature protection).

## 3 Results

### 3.1 Behavioural Response of All Wolves Towards Scent Marks

Each experimental station included three scent stimuli: unknown wolf urine, unknown dog urine, and water as a control, all placed in random order. In no case did wolves show any reaction to the water control, and no time was recorded for sniffing or marking it (0 s across all trials). A summary of mean sniffing durations across wolf groups and scent stimuli is presented in Table 2.

**TABLE 2:** Mean duration of sniffing behaviour (in seconds) ± SE across wolf and scent stimuli.

Wolf group	Mean sniffing duration (s) ± SE
All wolves (DOG)	14.2 ± 3.7
All wolves (WOLF)	48.3 ± 11.5
All wolves (overall)	59 ± 9.28
Juveniles (DOG)	7.1 ± 3.4
Juveniles (WOLF)	37.1 ± 8.9
Juveniles (overall)	44.1 ± 9.2
Breeding pair (overall)	26.3 ± 8.3
Breeding pair (DOG)	7 ± 1
Breeding pair (WOLF)	28.8 ± 8.2
Breeding male (overall)	14.4 ± 3.7
Breeding female (overall)	11.4 ± 3.4
Mean juvenile (overall)	12.5 ± 2.3
Mean juvenile (DOG)	16 ± 2.7
Mean juvenile (WOLF)	7.3 ± 3.3

Abbreviations: Dog, unknown dog; Wolf, unknown wolf.

In total, wolves reacted to the presented scent stimuli in 55 independent behavioural events. The majority of responses were directed towards unknown wolf urine (*n* = 41), followed by unknown dog urine (*n* = 14). These counts include all recorded events of sniffing, overmarking, or ground scratching directed towards the specific stimulus. When wolves were recorded at our experimental locations, in 59.6% of all cases, they showed a behavioural response to our experimental scent marks. When wolves displayed a response to scent marks, they spent the most time sniffing the stimuli, for an average of 59.0 ± 9.28 s of visit duration. They spent, on average, 14.2 ± 3.7 s of visit duration sniffing unknown dog stimuli (*F*_2,36_ = 12.05; *p* < 0.001). However, wolves concentrated mainly on sniffing unknown wolf stimuli, on which they spent 48.3 ± 11.5 s of visit duration.

### 3.2 Behavioural Response of Breeding Pair and Juvenile Wolves

The responses of the breeding pair members and juveniles to scent marks differed in both duration and behavioural expression. Juveniles exhibited only one type of response: sniffing. However, the duration of their sniffing differed significantly between unknown dog and unknown wolf scent marks (*F*_2,36_ = 9.278; *p* < 0.001). In total, juveniles spent 441 s sniffing, which constituted an average of 60.9% of the time they were recorded in front of the cameras. They spent more time sniffing scent marks from unknown wolves than from unknown dogs (32.2 ± 1.8 s vs. 20.3 ± 2.5 s, respectively). The remaining 39.1% of their time was devoted to behaviours unrelated to the scent marks (Table 3A, Figure 2).

**TABLE 3:** Comparison of the effect of scent stimuli on the behaviour of breeding (A) pair members and offspring in KNP, using the Tukey‐HSD test, (B) females, breeding males, and juveniles in KNP, using a Tukey‐HSD test.

Individuals	Behaviour	Scent stimuli	95% confidence level		Level of statistical significance
			Lower	Upper	
(A)					
Breeding pair members	Sniffing	WOLF—DOG	−0.1006761	20.100676	0.0527940*
		Water—DOG	−11.177599	9.023753	0.9632908
		Water—WOLF	−21.177599	−0.976247	0.0289758*
	Overmarking	WOLF—DOG	−1.153465	2.0765418	0.7658653
		Water—DOG	−2.076542	1.1534649	0.7658653
		Water—WOLF	−2.538080	0.6919264	0.3530720
	Ground scratching	WOLF—DOG	−1.153465	2.0765418	0.7658653
		Water—DOG	−2.076542	1.1534649	0.7658653
		Water—WOLF	−2.538080	0.6919264	0.3530720
Juveniles	Sniffing	WOLF—DOG	5.858268	40.14173	0.0063829***
		Water—DOG	−22.603270	11.68019	0.7182921
		Water—WOLF	−45.603270	−11.31981	0.0007272***
(B)					
Breeding female	Sniffing	WOLF—DOG	−0.3596681	7.89812968	0.0793981
		Water—DOG	−4.4365912	3.82120661	0.9818838
		Water—WOLF	−8.2058220	0.05197584	0.0535511*
	Overmarking	WOLF—DOG	−0.592380	1.3616108	0.6050766
		Water—DOG	−1.207765	0.7462262	0.8330087
		Water—WOLF	−1.592380	0.3616108	0.2848573
	Ground scratching	WOLF—DOG	−3.731064	9.423371	0.5460422
		Water—DOG	−8.192602	4.961833	0.8208208
		Water—WOLF	−11.038756	2.115679	0.2351357
Breeding male	Sniffing	WOLF—DOG	0.8444258	11.617113	0.0203164*
		Water—DOG	−6.1555742	4.617113	0.9351602
		Water—WOLF	−12.386343	−1.613657	0.0083771**
	Overmarking	WOLF—DOG	−0.5833915	−0.9680069	0.9563428
		Water—DOG	−0.8910838	0.4295453	0.6721523
		Water—WOLF	−0.9680069	0.3526223	0.4968281
	Ground scratching	WOLF—DOG	−3.206934	8.130011	0.5437635
		Water—DOG	−6.360780	4.976165	0.9521262
		Water—WOLF	−8.822319	2.514626	0.3722552
Juvenile	Sniffing	WOLF—DOG	−2.195694	13.580309	0.1961337
		Water—DOG	−13.349540	2.426463	0.2219208
		Water—WOLF	−19.041847	−3.265845	0.0039673**

*Note:* Asterisks indicate levels of statistical significance: *p < 0.05, **p < 0.01, *** *p* < 0.001.

Abbreviations: Dog, unknown dog; Wolf, unknown wolf.

Members of the breeding pair spent 255 s reacting to all the scent marks, which was 61% of the time they were recorded in front of the cameras. The breeding pair members spent the longest time sniffing (average 26.3 ± 8.3 s). The “unknown wolf” stimuli were sniffed longer than the “unknown dog” stimuli (on average 28.8 ± 8.2; 7 ± 1, respectively). The breeding pair's second longest behavioural response to scent marks was ground scratching, which comprised 19.7 ± 6.0 s of visit duration; wolves spent on average 19.3 ± 8.4 s ground scratching in response to the unknown wolf stimulus. The unknown dog stimulus was scratched only once for 21 s. The difference between these values was not statistically significant (*F*_2,36_ = 1.409; *p* > 0.05). Wolves spent the least time (on average 1.7 ± 1.1 of visit duration) on overmarking the stimuli, spending an average of 6.0 ± 2.0 s overmarking the unknown wolf stimuli. The unknown dog stimulus was overmarked only once, and the behaviour lasted 6.0 s (Table 3A, Figure 2). Juveniles (all individually scored combined individuals) spent 441 s sniffing stimuli, which accounted for an average of 19.2 ± 2.5 s of visit duration. The difference between the times they spent sniffing unknown dog and unknown wolf stimuli was statistically significant (*F*_2,36_ = 9.278; *p* < 0.001).

### 3.3 Behavioural Response of Male, Female, and Average Juvenile

Breeding males spent more time (66% of visit duration) reacting to scent marks than carrying out behaviours not aimed at the scent marks (34% of visit duration). Breeding male wolves spent the main part of their time sniffing scent marks (on average 14.4 ± 3.7 s), followed by scratching the ground (on average 9 ± 4.7 s). He spent the least amount of time overmarking stimuli (on average 1.0 ± 0.5). The difference between the times he spent sniffing unknown dog and unknown wolf stimuli was statistically significant (*F*_2,36_ = 6.069; *p* < 0.01). For both overmarking and ground scratching, there were no differences between the times spent reacting to the unknown dog and unknown wolf stimuli (respectively, *F*_2,36_ = 1.21; *p* > 0.05 and *F*_2,36_ = 1.409; *p* > 0.05) (Table 3B and Figure 2B).

Breeding females spent 54% of the time female wolves were recorded on camera traps responding to stimuli, and 46% carrying out behaviours unrelated to the stimuli. The female spent the most time sniffing stimuli (on average 11.4 ± 3.4 s), then scratching the ground (on average 5.8 ± 2.4 s). The female spent the least time overmarking, which constituted, on average, 2.2 ± 0.9 s of visit duration. The difference between the time spent sniffing unknown wolf and unknown dog stimuli was statistically significant (*F* = 3.612; *p* < 0.05); however, the results of the Tukey HSD test were not statistically significant (Table 3B and Figure 2B). For overmarking and ground scratching, there were no differences between the times spent reacting to unknown wolf and unknown dog urine (respectively, *F*_2,36_ = 0.703; *p* > 0.05 and *F*_2,36_ = 1.022; *p* > 0.05).

“Mean juvenile” spent 60% of the visit duration sniffing stimuli, which accounted for on average 12.5 ± 2.3 s. The single juvenile sniffed unknown wolf stimuli for longer than unknown dog stimuli, and this difference was statistically significant (*F*_2,36_ = 5.974; *p* < 0.01) (Table 3B and Figure 2B).

## 4 Discussion

The recovery of carnivore populations in Europe (Chapron et al. 2014; Kuijper et al. 2016) has created a situation where wolves and dogs increasingly share a landscape in which they can potentially interact (Hughes and Macdonald 2013; Lescureux and Linnell 2014). This raises questions about whether they interact and the ecological consequences of these interactions. Our results show that wolves display a clear behavioural response to dogs. We acknowledge that this study is preliminary, being confined to a single forest complex and involving only two wolf groups. This limited scope may affect the generalizability of our results, as wolf responses to dog scent marks could vary across different habitats. It is possible that the unique context of Kampinos National Park may not necessarily reflect broader trends. Nonetheless, whether this situation is exceptional or representative of interactions in other forest ecosystems, our study serves as an important initial exploration of this behaviour, as wolves investigated dog scent marks 14 times during the experiment. When they responded, they spent most of their time sniffing, showing that dog scent marks significantly affect wild dog behaviour. Wolves reacted less intensively (14 occurrences to 41 occurrences, respectively) to the scents of unknown dogs than to those of unknown wolves. Wolves sniffed the stimuli from unknown wolves for significantly longer than they did those from unknown dogs. The time spent overmarking and ground scratching did not significantly differ between the stimuli. Wolves' interest in the scents of unknown wolves, which they expressed by sniffing them for a long time, may be related to the defence of their territory against potential intruders of the same species (Bekoff 2001). That wolves spent a significantly shorter time sniffing the dog stimuli could suggest they distinguish between dog and wolf urine marks and perceive unknown dogs as less of a threat than unknown wolves. However, if that is true, we could also expect that wolves, in response to the dog stimuli, will spend less time on overmarking and ground scratching. Both these behaviours are related to the defence of territories, when performed by the members of a breeding pair (Peters and Mech 1975; Dunbar 1977; Allen et al. 1999; Pal 2003; Zub et al. 2003; Stępniak et al. 2020). A potential explanation for this is that wolves spent less time sniffing the stimulus from the unknown dog due to the frequent presence of dogs in KNP (Diserens et al. unpublished data). This may have resulted in a form of “scent pollution” effect that disrupts the natural behaviour of wolves. If this is the case, a new experiment in a place with lower anthropopressure and fewer dogs should find that wolves sniff stimuli from the unknown dog and wolves for similar amounts of time. Young wolves spent more time (7.3 ± 3.3; 1.4 ± 0.8; 0.8 ± 0.8 s for “mean juvenile”; breeding male; breeding female respectively) exploring dog urine compared to adults. They also spent similar amounts of time sniffing both stimuli. A possible explanation for this behaviour is that they are less able to distinguish between the scent of dogs and wolves.

The interest wolves have in the scents of conspecifics, as well as their reactions to them in the form of overmarking, are aimed at preventing aggressive interactions between wolf packs, which reduces their likelihood of being injured or killed (Gosling 1982). However, the scent marking process is costly, so wolves mark only the most important parts of their territories (Zub et al. 2003) and intensify marking during particularly sensitive periods, for example, during heat (Peters and Mech 1975; Zub et al. 2003) and while caring for pups (Llaneza et al. 2014). Moreover, wolves choose places where their markings can easily be detected by other individuals (Barja et al. 2004; Stępniak et al. 2020). In this context, all activities aimed at protecting the territory performed by wolves in KNP should result in intruders withdrawing from their territory. However, the persistent occurrence of dogs in KNP shows that the wolves' response to dog scent marks does not have the intended effects. Most dogs in KNP, regardless of whether they are on a leash or not, are under human control, which limits their natural behaviour and to a large degree determines where they will move. Thus, wolves overmarking dog scent markings cannot cause dogs to stop appearing. As a result, wolves spend valuable energy resources on a behaviour that only has limited or no benefit.

The average time spent responding to the dog scent stimulus was 7.3 ± 3.3 s (mean juvenile); 1.4 ± 0.8 s (breeding male); 0.8 ± 0.8 s (breeding female). During the study period, dogs were recorded 91 times, which means that if each of these dogs left one scent mark per visit, to which later passing wolves reacted with a similar frequency as to the stimuli in the present study, the juveniles would spend 664 s, and the breeding pair 127 s and 72 s (male and female, respectively) only responding to these stimuli. These calculations do not consider other factors, such as canines being able to distinguish between odours repeatedly left by the same individuals, and therefore react to them less with each subsequent contact (Brown and Johnston 1983). However, since this knowledge originates from studies on captive animals, it is difficult to assess whether such habituation also occurs in natural conditions. The hypothesis put forward by Gosling (1982) assumes that territory owners react with a similar commitment every time they come across a scent marking, as their main goal is to remove the smell of a foreign individual from their territory.

The importance of the interactions between wolves and dogs that occur via scent marking has so far been underexplored. The most frequently discussed aspects of the interactions between these two predators are hybridization, parasite transmission, and competition for resources (Lescureux and Linnell 2014); hence, it is difficult to compare our results with previous studies. However, in contrast with the main result of our study, a previous experiment found that dingoes ( *Canis dingo* ) reacted more intensely to the scents of domestic dogs than to those of their own species. However, the authors assumed that this reaction was related to the tested dingoes already being familiar with the smell of the dingoes whose urine was used in the experiment (Van Bommel and Johnson 2017). Research investigating the use of predator scents in the protection of livestock (biofencing) found that in the second year of the experiment, wild wolves responded less intensively to the scent of unknown wolves than in the first year. The authors explained that this result was due to the scent stimuli being placed in the wolves' territory for only short discontinuous periods, which may have meant that the resident wolves may perceive discontinuous periods during which the ‘intruder’ wolves were absent in the next year (Ausband et al. 2013). However, in KNP, where dogs are present year‐round, it seems unlikely that such a mechanism would be possible.

Wolves living in KNP reacted to the stimuli from unknown wolves and dogs, which confirms that territorial wolves react to the scents of unfamiliar individuals (Peters and Mech 1975). The smell from an unknown individual is a source of important information about potential partners or threats (Lisberg and Snowdon 2009). However, we did not expect the juveniles to respond for a longer time than their parents to both scent stimuli. The literature has assumed that mainly breeding individuals are responsible for defending territories against intruders (Peters and Mech 1975); however, recent research has shown that juveniles also take part by marking the area with their own scent (Stępniak et al. 2020). Our results, particularly the difference between the length of reaction to stimuli between adult and juvenile individuals, suggest that juvenile wolves may learn from their parents when travelling through the territory with them. Thus, the presence of dogs in wolf territories may obstruct the process of learning.

The analysis of sex‐based differences in behavioural responses was included to explore potential patterns, but given the limited sample size, these results should be interpreted as preliminary. They may reflect individual variation rather than true sex‐related differences. Future studies with larger sample sizes are necessary to validate whether consistent behavioural differences exist between male and female wolves in response to scent marks.

### 4.1 Are Dogs a Threat to Wolves?

Wolves are recolonizing landscapes intensively used by humans and their dogs, meaning that interactions between wolves and dogs will become more frequent. We assume that most of these encounters will be indirect; our results indicate that wolves and dogs communicate, and that this can influence wolf behaviour. Based on our results, it is not possible to determine whether wolves regard dog scents as a threat signal or a harmless distraction. Adult wolves only explored “unknown dog” scents briefly, which could suggest that they do not see them as a threat. On the other hand, they overmarked them with the same intensity as the “unknown wolf” scent, signalling to the intruder that the territory is already occupied by a group of wolves. Therefore, further studies are required to determine if dog scent negatively impacts wolf territorial behaviour.

Our study area is under strict and active protection, except for assistance dogs. However, a camera trap monitoring survey (Diserens et al., unpublished data) found that dogs were the most abundant predator in KNP, among wolves, lynx, foxes, and badgers. If wolves living in this area react to even a small number of dog markings, we can assume that this is more distracting than in a forest with fewer dog visits. This means that the presence of dogs in wolf territories may have consequences for wolf natural behaviour, and therefore, more emphasis should be placed on exploring the consequences of this situation.

Our findings contribute to the understanding of behavioural divergence between wolves and dogs, particularly in the context of chemical communication and territoriality. The different intensity and nature of wolf reactions towards dog scent marks compared to conspecific scent marks indicate a potential divergence in communication patterns that may have developed during the domestication of dogs. While wolves clearly prioritise scent marks from conspecifics, suggesting strong territorial motivations (Barja et al. 2004; Peters and Mech 1975; Stępniak et al. 2020; Zub et al. 2003), their relatively brief attention to dog scent marks may reflect an adaptation to frequent, non‐threatening encounters with domestic animals (Bekoff 2001). These behavioural differences provide valuable insights into the evolutionary processes underlying behavioural divergence between domesticated dogs and their wild ancestors.

## Author Contributions

**Kinga M. Stępniak:** conceptualization (equal), data curation (equal), formal analysis (equal), methodology (equal), visualization (equal), writing – original draft (equal), writing – review and editing (equal). **Tom A. Diserens:** data curation (equal), writing – review and editing (equal). **Maciej Szewczyk:** data curation (equal), writing – review and editing (equal). **Robert W. Mysłajek:** data curation (equal), supervision (equal), writing – review and editing (equal). **Dries P. J. Kuijper:** conceptualization (equal), methodology (equal), resources (equal), supervision (equal), writing – review and editing (equal).

## Conflicts of Interest

The authors declare no conflicts of interest.

## Data Availability

Data used in this study may be obtained on the website https://zenodo.org/records/12187601.
